# Design of Modern Reactors for Synthesis of Thermally Expanded Graphite

**DOI:** 10.1186/s11671-015-0919-y

**Published:** 2015-05-29

**Authors:** Eugene V. Strativnov

**Affiliations:** Gas Institute of the National Academy of Sciences of Ukraine, Kyiv, 39, Degtyarivska str., 03113 Kyiv, Ukraine

**Keywords:** Thermally expanded graphite, TEG, Graphene, Absorbent, Thermal shock, Energy efficiency, Simulation, CFD, ANSYS, SolidWorks, Computational methods in fluid dynamics, 47.11.-j, Structural classes of nanoscale systems, 62.23.-c, Carbon/carbon-based materials, 81.05.U-

## Abstract

One of the most progressive trends in the development of modern science and technology is the creation of energy-efficient technologies for the synthesis of nanomaterials. Nanolayered graphite (thermally exfoliated graphite) is one of the key important nanomaterials of carbon origin. Due to its unique properties (chemical and thermal stability, ability to form without a binder, elasticity, etc.), it can be used as an effective absorber of organic substances and a material for seal manufacturing for such important industries as gas transportation and automobile. Thermally expanded graphite is a promising material for the hydrogen and nuclear energy industries. The development of thermally expanded graphite production is resisted by high specific energy consumption during its manufacturing and by some technological difficulties. Therefore, the creation of energy-efficient technology for its production is very promising.

**Electronic supplementary material:**

The online version of this article (doi:10.1186/s11671-015-0919-y) contains supplementary material, which is available to authorized users.

## Background

Systematic research of the methods of thermally expanded graphite (TEG) synthesis and their application have emerged about the last 25–30 years [1].

### The Stages of Obtaining TEG

**Fig. 1:**
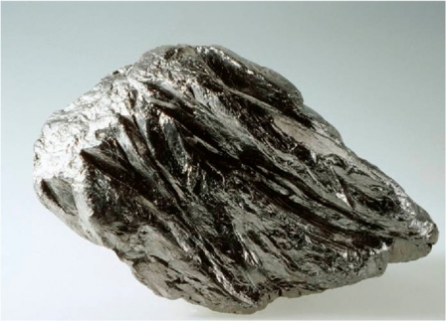
The view of natural crystalline graphite

The raw material of TEG is natural crystalline flake graphite (Fig. 1). Deposits of flake graphite are situated in Ukraine (Zavalie, Kirovograd region), China, Russia, and the USA. The great manufacturers of graphite are China, India, Korea, and Brazil.

In the first stage of TEG synthesis, natural crystalline graphite is crushed into flakes and oxidized [2]. Oxidation is the impregnation of molecules and ions of sulfuric or nitric acid in to the layers of the graphite’s crystal lattice in the presence of an oxidant (hydrogen peroxide, potassium permanganate, or others). This process is called intercalation. The obtained material is called oxidized graphite (OG).

**Fig. 2:**
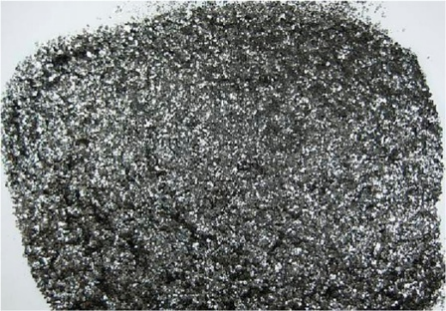
The view of intercalated flake graphite

Then it is washed with water to remove excess intercalant and dried to obtain its technological properties (see Fig. 2).

**Fig. 3:**
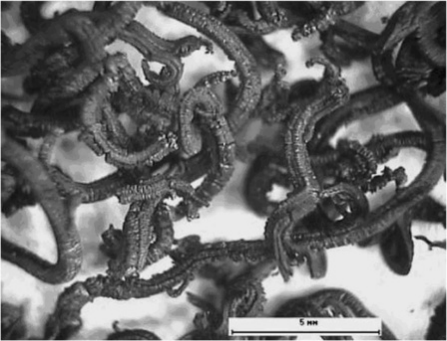
The view of TEG at the macro level

**Fig. 4:**
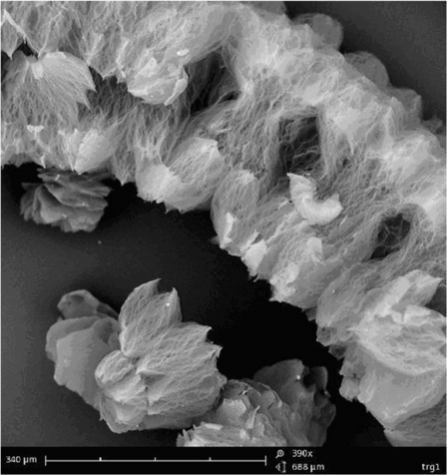
The view of TEG at the micro level

The next step is OG heating in a high-temperature reactor to the temperature of OG expansion. Due to the extremely high heating rate (600 °C/s), there is a sharp expansion of gaseous products of decomposition of the introduced sulfuric acid from the graphite’s crystal lattice. Thermally expanded graphite is obtained by increasing the interlayer distance in the scaly graphite (about 300 times). The view of TEG at the micro and macro levels is shown in Figs. 3 and 4.

A separate part of TEG is a piece of worm-like shape with a diameter of 0.1–0.5 mm and a length of 6–10 mm. This particle consists of a set of interconnected individual layers of graphite (graphene) and packs (10–50 PCs) of such layers.

### General Use of TEG

**Fig. 5:**
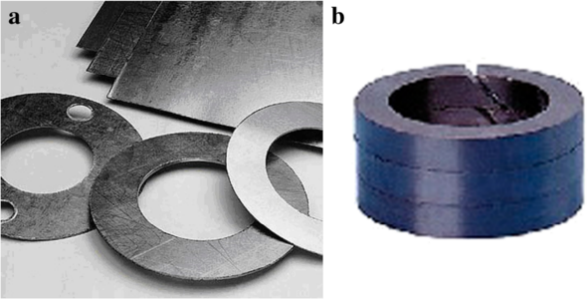
Products from TEG by rolling (**a**) and pressing (**b**)

Gaskets and seals can be manufactured from TEG (see Fig. 5) with unique properties by pressing and rolling without any binders and reinforcement [3, 4]. Products can be used in a wide temperature range and under the influence of aggressive environments.

**Fig. 6:**
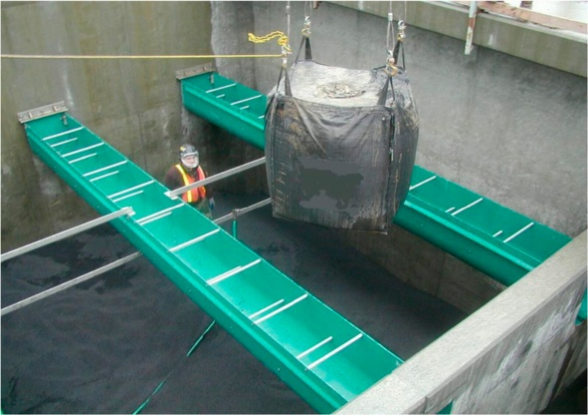
The use of TEG for water purification in wastewater treatment plants

Thus, sealing goods found their application in various engineering industries—from cryogenic to nuclear technologies. TEG is used as a component of batteries of new generation [5]. In the case of TEG addition to the cathode active mass of alkaline zinc-manganese dioxide batteries, the coefficient of utilization of the active material reaches up to 40 % vs. 29 % in the case of application of natural graphite. TEG is also a unique absorbent of such substances as helium, argon, nitrogen, krypton, hydrogen, xenon, isooctane, benzene, and cyclohexane. Basically, TEG is used as an absorber [6] of organic substances (see Fig. 6). Additional movie files show this in more detail [see Additional files 1, 2, and 3]. Due to its nature, TEG absorbs liquid organics not only by individual particles but also by clusters of its particles.

### Heating Methods of OG Particles

OG heating by heat from combustion of liquid or gaseous fuel is currently the most common method. It is realized in a furnace with a fluidized bed of inert heat carrier and in furnaces with a concurrent flow of flue gases with the target product. This technology was taken as the basis for a comprehensive study and analysis. The most important factor which affects TEG quality and energy consumption is the heating rate of OG. For intensification of heat exchange between OG particles and the flow of fuel combustion products, the turbulence in the reaction zone is increased. Also, OG feeding is accomplished directly into the core of the flame [7]. Additional movie files show this in more detail [see Additional files 4, 5, and 6]. Specially developed devices have complex geometry of the internal space (and not symmetric). Therefore, modeling of physical processes occurring inside such devices is proposed to be carried out by 3-D with the help of modern software systems (for example, ANSYS and SolidWorks) [8, 9]. In this manuscript, we considered two new types of reactors: a reactor with two opposite burners (one of which is a “burner-feeder”) and a reactor with a “cyclone-type” apparatus. Feeding of combustion products and OG into the reaction zone is carried out tangentially.

## Methods

**Fig. 7:**
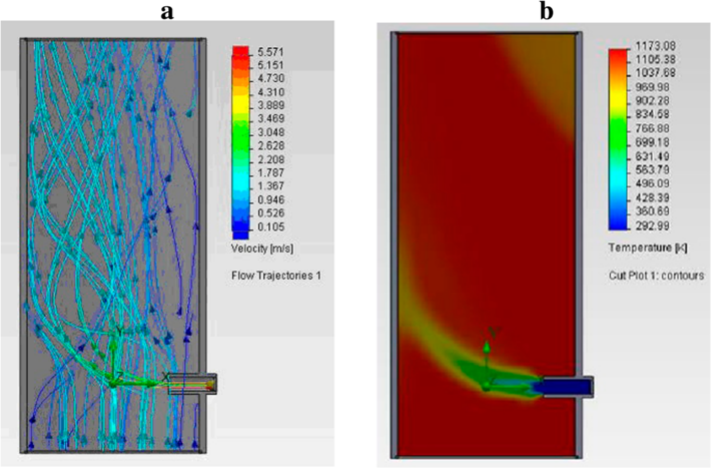
Gas flows (**a**) and their temperature (**b**) in the reactor for TEG synthesis by “the classical scheme”

The significant deficiency of the “classic” technological scheme was identified while analyzing the hydrodynamics of the flow and temperature field distribution inside the reactor. This disadvantage consists the following. Cold air is fed with raw materials into the reactor and dilutes by itself smoke gases and thereby lowers the temperature in the reaction zone (gas flows and their temperature are shown in Fig. 7). Flue gases with a higher temperature are not used it is not rational.

## Results and Discussion

### Choosing the Type of Apparatus and Calculation of Material Flows

It is shown that to improve TEG quality, it is necessary to increase the OG heating rate. As a result, we created a new technology for TEG production. The main idea of the proposed method is that the raw materials are fed into the reactor together with air, used for fuel combustion. Moreover, the gas dynamic characteristic of the two-phase flow is that particles of OG are fed directly into the core of the flame. OG particles are actively heated in the core of the torch, effectively using the convective and radiation components of heat transfer. A high rate of the convection component is implemented due to the reactor’s zone of turbulence and by the dynamic pulsations that occur during fuel combustion. High level of radiation component is due to the direct contact of the “glowing” flame and OG particles.

**Fig. 8:**
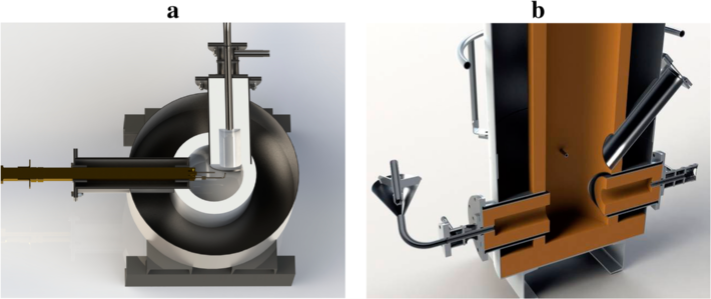
Reactors for the production of TEG: cyclone type (**a**) and opposite type (**b**)

A new technological scheme is implemented in two reactors: a reactor of “opposite type” and a reactor of “cyclone type” (see Fig. 8). In the cyclone-type reactor, the flow of combustion products is fed tangentially. Thanks to such feeding scheme, the product’s residence time inside the reactor increases while the dimensions of the device are reduced.

The expansion occurs in the burner-feeder at the same time the vertical section of the reactor is used for the final pre-expansion and additional annealing of TEG in order to remove residual compounds of the intercalant. Increasing the heating rate and the total reaction temperature allowed obtaining TEG of higher quality with a density of 3 g/l and higher “purity” of the product. These can extend the scope of the application of this material up to its application in the nuclear industry.

The design of the apparatus involves the calculation of its geometrical parameters. This calculation means the determination of its material flows. The calculations are carried out basing on the hover speed of OG particles (for OG pneumatic transportation) and TEG (for TEG removal from the working area of the reactor). Calculation of material flows is connected with the volume of flue gases produced from fuel combustion and the desired performance of the aggregate. According to these calculations, the source material (OG) must be guaranteed to be delivered in a high-temperature zone of the reactor and the finished product (TEG) has to leave it due to the speeds of the appropriate gaseous flows.

### Study of Hydrodynamics in Reactors of New Type

**Fig. 9:**
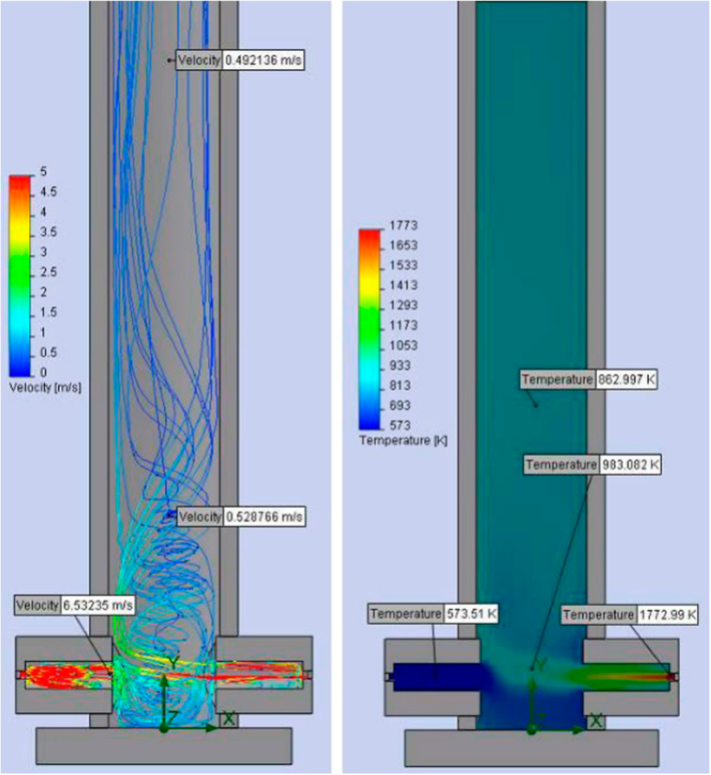
The mixing process of counter-flows in the reactor of opposite type

New technology has allowed a much better use of the heat from fuel combustion, which has a positive impact on the values of specific energy consumption. In particular, for the apparatus of opposite type as shown in Fig. 9, due to the active mixing of the gas streams at the bottom of the reactor, there is almost complete equalization of temperature throughout the volume in the vertical part of the apparatus. In this particular case, to illustrate the mixing process, cold air was fed.

**Fig. 10:**
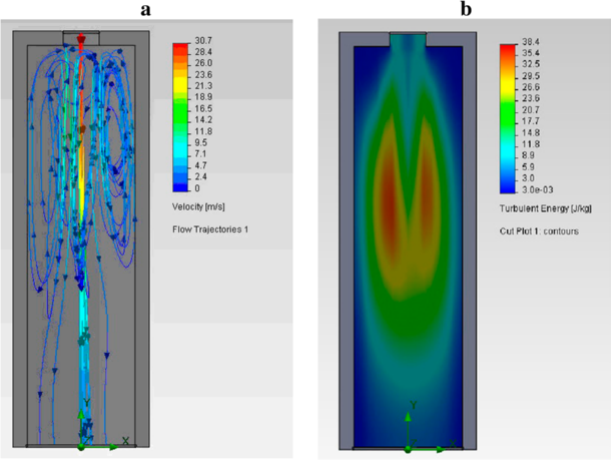
Trajectory and velocity of gas flows (**a**) and their corresponding distribution of turbulence energy (**b**) in the eddy zone of the burner

Figure 9 shows the data after the complete cessation of combustion in the burner-feeder. That is in the most unfavorable (emergency) time of apparatus operation. Operating mode (when both burners are working) knowingly provides better gas mixing and temperature equalization in the reactor. The height and diameter of the reactor are designed to ensure the continuous (about 15 s) stay of the material in the working zone during the “working” regimes of the apparatus. Longer stays of TEG in the high-temperature zone can additionally calcinate the final product. The size of the turbulence zone in the tunnel burner is designed for active mixing of hot gases and to form cyclical flows (see Fig. 10). This hydrodynamic regime provides autoignition of feeding mixture and combustion stabilization.

**Fig. 11:**
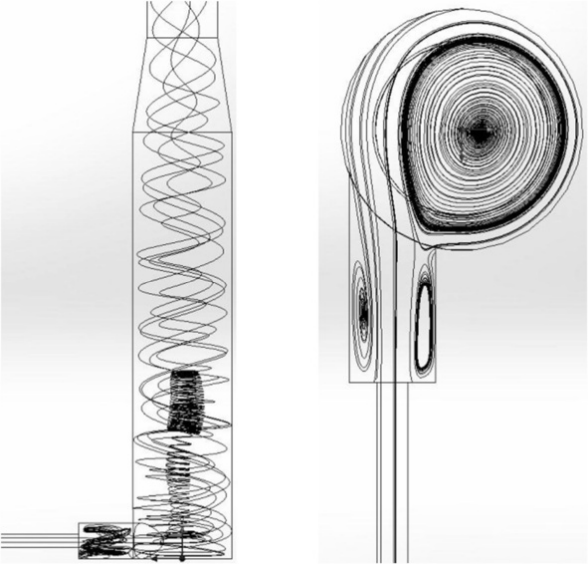
Flows in the longitudinal (*left*) and transverse (*right*) cross sections of the cyclone-type aggregate

The eddy zone of the burner is identical to the eddy zone of the burner-feeder of the TEG generation apparatus, where the direct contact of OG and hot gases occurs. The turbulence energy in the eddy zone is 35–38 J/kg, which provides a significant (30-fold) increase of the thermal conductivity of the flue gases (1.95 W/m·K at 1000 °C) compared to their conductivity at the quiescent mode (0.0667 W/m·K at 1000 °C). In the reactor of cyclone type, due to swirling of a flow, TEG residence time increases at relatively small dimensions of the device (Fig. 11).

### Simulation of Methane Combustion

The process of fuel combustion contributes additional ripples to flow hydrodynamics. In this regard, the heat exchange between OG particles and the flame has some nuances. The formal result of the modeling of this process is the calculation of the final temperature and the heating rate of OG particles. However, during the modeling process, information and additional knowledge about the behavior of the investigated material appear, for example, the magnitude and the direction of particle trajectories, the Reynolds criterion for particles, and time of staying in the reactor. All this information brings researchers to the next step closer to the understanding of the processes occurring with the investigated material.

**Fig. 12:**
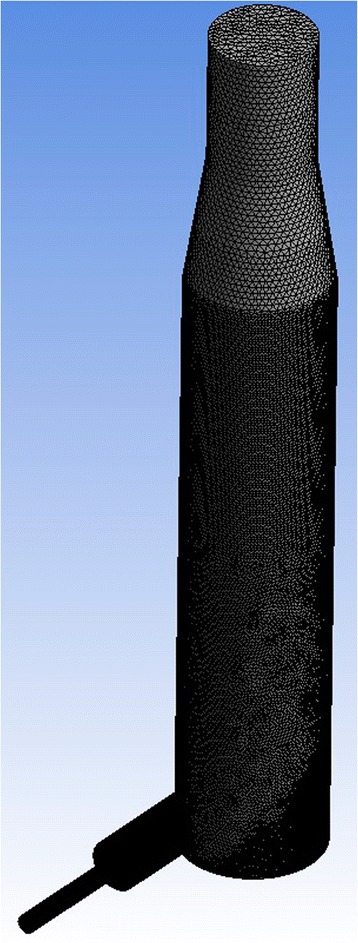
Workspace of a cyclone-type apparatus

**Fig. 13:**
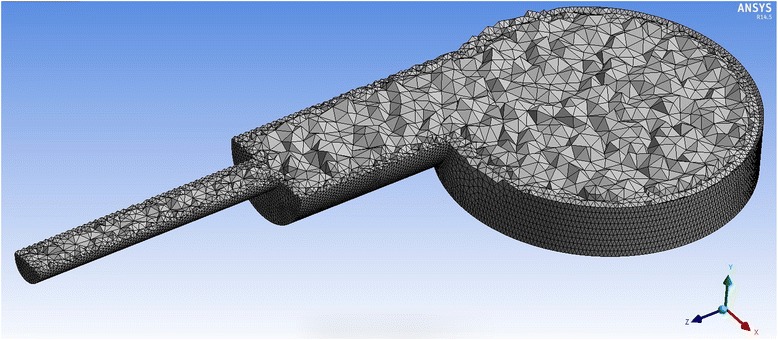
Cross section of the active area of a cyclone-type apparatus

The simulation includes several stages: construction of the geometry of the model, calculation of grid construction, setting of source data, choosing of a solver, and solution of the task. To ensure the sufficient accuracy of the solution, the workspace in our case is divided into 1,028,519 cells of the grid (which corresponds to 1,473,273 nodes of a hexagonal grid). The grid (see Figs. 12 and 13) has a non-uniform break step to improve the accuracy of the calculation in the most “intense” sections of the space of the reaction zone. The grid is usually deliberately compacted in areas where there has been an increase in comparison with the rest of the space for heat and mass transfer and also at the walls of the apparatus in order to account for the near-wall effects of hydrodynamic flow.

Modeling of the process of methane combustion is not the purpose of this work, but, because of its complexity, a more detailed description is required, at least the original data and the applied models and methods. The author supposes that this information will be useful for professionals who engage in simulation by ANSYS. Table 1 presents the specific coefficients.

**Table 1:** Coefficients used for the design of the process of burning of methane

No.	Reagents			Products			Arrhenius rate			Mixing rate	
		Stoich. Coeff.	Rate exp		Stoich. Coeff.	Rate exp	Pre-exp factor	Activation energy	Temperature exponent	A	B
1	CH_4_	1	1.46	CO	1	0	1.66e15	1.7e8	0	4	0.5
	O_2_	1.5	0.5217	H_2_O	2	0					
2	CO	1	1.7	CO_2_	1	0	7.9e14	9.6e7	0	4	0.5
	O_2_	0.5	1.57								
3	CO_2_	1	1	CO	1	0	2.2e14	5.2e8	0	4	0.5
				O_2_	0.5	0					
4	N_2_	1	0	NO	2	0	8.8e23	4.4e8	0	1e11	1e11
	O_2_	1	4.01								
				CO	0	0					
	CO	0	0.72								
5	N_2_	1	1	NO	2	0	9.27e14	5.7e8	−0.5	1e11	1e11
	O_2_	1	0.5								

The parameters of the solver used to simulate the gas-thermal flow of methane combustion products are as follows:

- Turbulence model—k-epsilon (2 eqn)
- Submodel—realizable
- Option, considering the parietal effects—“near-wall treatment” (standard wall function)
- Radiative transfer model—“P-1” model
- Wavelength intervals—band 0 = 2.8–4.11, band 1 = 4.5–8.76

**Fig. 14:**
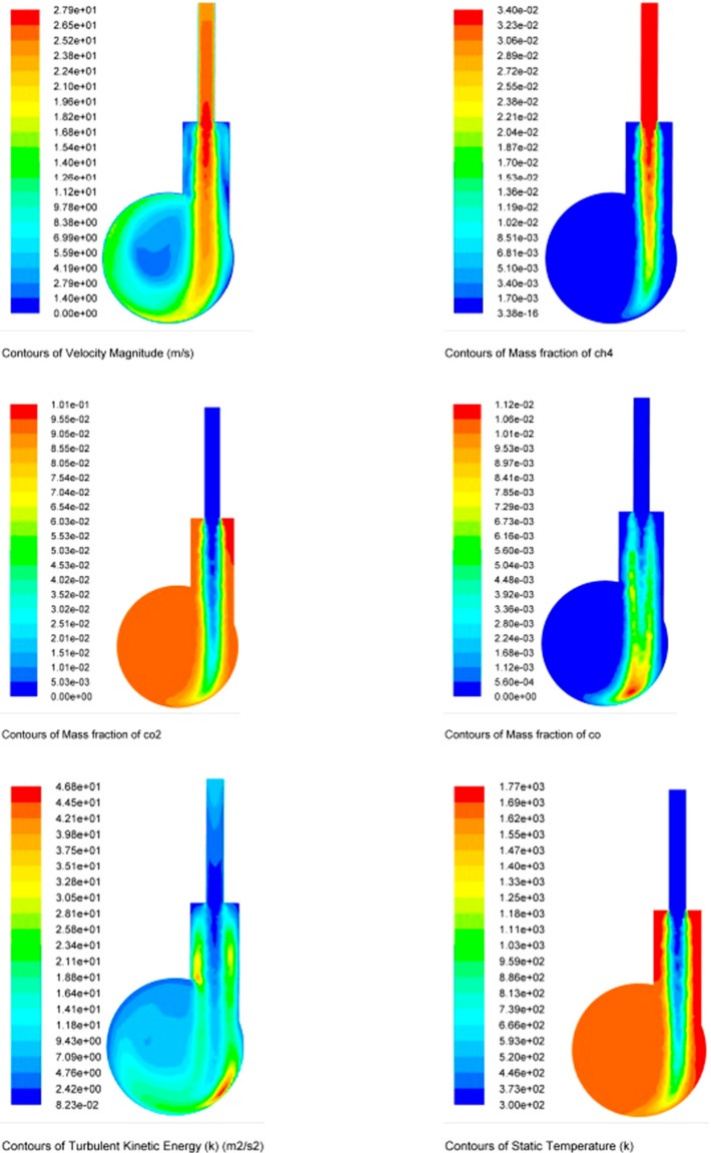
Plots of flow rate distribution, gas concentrations, levels of energy turbulence, and temperatures in the cross section of a cyclone-type reactor

The physical conditions of the flow of OG particles are as follows: the distribution of the temperature fields, the composition of combustion products, etc. (Fig. 14). Modern modeling tools and analysis (in this case, ANSYS software) allow, also, to operate with massive OG particles of different size distributions, to determine their absolute and relative velocities, to determine the Reynolds criterion, etc.

### Investigation of the Heating Rate of OG Particles in the New-Type Reactor Considering the Hydrodynamic Pulsations that Occur During Methane Combustion

**Fig. 15:**
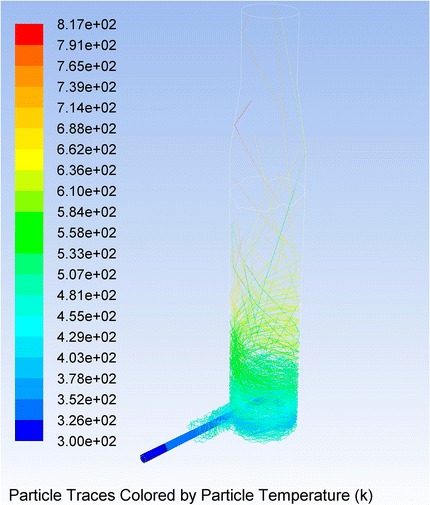
Trajectory and temperature of OG particles in a cyclone-type reactor

The oxidized graphite particles carried by a stream of hot products of combustion of methane are instantly heated up to study the heating rate of the particles of OG in conditions as close as possible to the real, simulated flow rates in the reactor (see Fig. 15).

Solver’s parameters used to simulate the behavior of OG particles in the flow of products of methane combustion are as follows:

- Volumetric
- Diffusion energy source
- Mixture material: methane-air-2-step; number of volumetric species = 7
- Properties of methane-air-2-step: mixture species: CH_4_, O_2_, CO_2_, CO, H_2_O, NO, N_2_
- Density—incompressible ideal gas
- Heat capacity—using the law of mixing environment
- Thermal conductivity = 0.0241 W/m·K
- Viscosity = 1.72е−5 kg/ms
- Mass diffusion = 2.88е−5 m^2^/s
- Turbulence-chemistry interaction—finite rate/eddy dissipation

The following forces, acting on OG particles in the gas flow, are considered in the simulation:

- Shape factor
- Particle radiation interaction
- Thermophoretic force
- Brownian motion
- Saffman lift force
- Virtual mass force
- Pressure gradient force

The source data for OG particles are as follows:

- Equivalent diameter of the particles, *D* = 0.43 mm
- Consumption of oxidized graphite, *G* = 30 kg/h = 0.0083 kg/s
- Initial temperature of the particles, *T* = 300 K
- Density, *ρ* = 2230 kg/m^3^
- Heat capacity, Cp = 1680 J/kg·K
- Thermal conductivity = 0.33 W/m·K

### Design of Modern Reactors for TEG Synthesis

Modern design and construction of an apparatus of a new type involve several basic steps: the selection of a unit type, calculation of material flows, sizing of the reactor and of the diameters of all pipes, checking the calculation of the rate of OG heating with the help of computer simulation, and finally designing by using appropriate software. Connecting sizes of the reactor’s details are chosen, if it is possible from standard range, to ensure seamless interfacing with adjacent equipment.

**Fig. 16:**
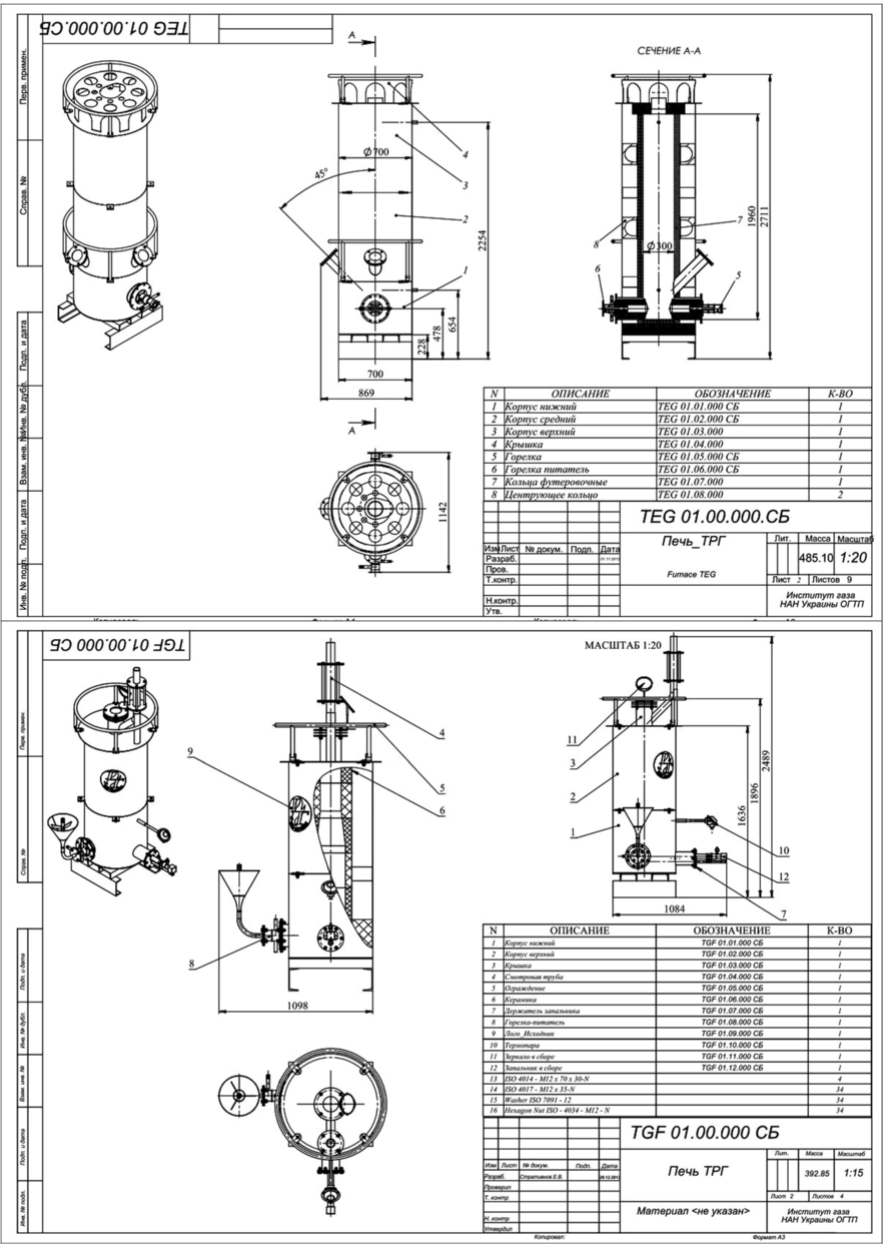
Assembly drawings of the new-type apparatus for TEG production

**Fig. 17:**
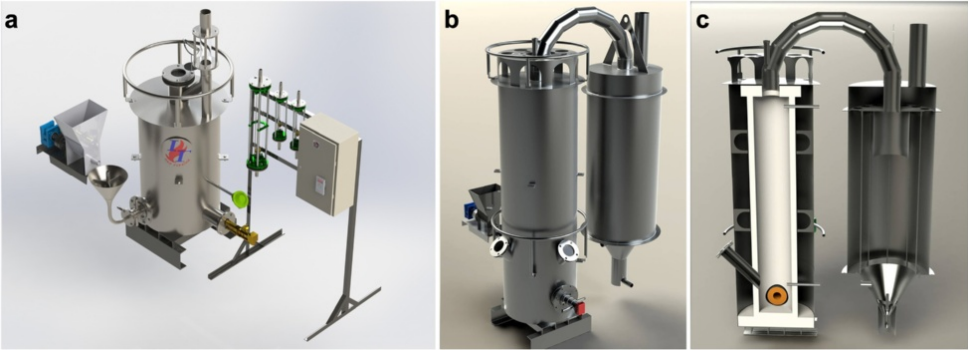
Computer visualization of the new-type systems for TEG production. Cyclone-type reactor (**a**) and opposite-type reactor (**b**, **c**)

On the order of Argonne National Laboratory (USA), a highly efficient apparatus of new type (opposite type) for TEG synthesis was developed and manufactured at the Gas Institute of the National Academy of Sciences of Ukraine in 2013. In 2015, it is planned to introduce the next apparatus of new type (cyclone type) at one of Ukraine’s plants. Assembly drawings of the new apparatus are presented in Figs. 16 and 17.

## Conclusions

Modern requirements for energy efficiency and resource saving, as well as a decrease of the weight of aggregates, require a more careful study of the processes occurring in the reactor and accurate calculations of the design and construction of units of a new type. For these purposes, it is appropriate to use the modern methods of numerical computer simulation in the field of computational fluid dynamics (CFD). Thus, energy efficiency during TEG generation and resource saving at equipment manufacturing for its production can be achieved.

## Additional files

Additional file 1:
**Water purification by TEG emulsion.** Demonstrates the efficacy of TEG. Additional file 2:
**Sand cleaning by TEG.** Demonstrates the efficacy of TEG. Additional file 3:
**Purification of the wing from oil by TEG.** Demonstrates the efficacy of TEG. Additional file 4:
**Start of TEG reactor.** Demonstrates the operation of the apparatus. Additional file 5:
**Hot TEG burner.** Demonstrates the operation of the apparatus. Additional file 6:
**Synthesis of TEG.** Demonstrates the operation of the apparatus.

## Abbreviations

TEG: thermally expanded graphite
OG: oxidized graphite
CFD: computational fluid dynamics
